# Post-burn Cervicofacial Necrotizing Fasciitis With Diabetic Ketoacidosis: A Report of a Case With a Favorable Outcome

**DOI:** 10.7759/cureus.53219

**Published:** 2024-01-30

**Authors:** Jasleen Kakkad, Shraddha Jain, Venkat Reddy, Keyur Saboo, Suhit Naseri

**Affiliations:** 1 Otolaryngology - Head and Neck Surgery, Jawaharlal Nehru Medical College, Datta Meghe Institute of Higher Education and Research, Wardha, IND; 2 General Medicine, Jawaharlal Nehru Medical College, Datta Meghe Institute of Higher Education and Research, Wardha, IND; 3 Medicine, Jawaharlal Nehru Medical College, Datta Meghe Institute of Higher Education and Research, Wardha, IND; 4 Pathology, Jawaharlal Nehru Medical College, Datta Meghe Institute of Higher Education and Research, Wardha, IND

**Keywords:** diabetes type ii, cervicofacial, diabetic ketoacidosis (dka), post-burn necrotizing fasciitis (pbnf), abscess

## Abstract

Post-burn necrotizing fasciitis (PBNF) is a serious and potentially life-threatening infection that occurs after a burn injury. It is characterized by rapid destruction of soft tissue and muscle and is usually caused by a bacterial infection. Diabetic ketoacidosis (DKA) is another serious complication of diabetes, which can occur when the body does not have enough insulin to break down glucose for energy. This causes the body to start breaking down fat for energy instead, leading to various complications. The present study discusses the association between PBNF and DKA in a patient with diabetes. Here is a case of a post-auricular abscess and a precipitated DKA. The abscess was located near the site of the previous burn injury that happened 20 years ago and was believed to have developed as a result of thick scar tissue. The patient was given adequate hydration, intravenous antibiotics, and insulin therapy. However, the abscess continued to grow with increasing insulin requirements and the patient underwent incision and drainage to remove the infected tissue, and an aggressive debridement was carried out. Thus, this case highlights the importance of closely monitoring blood sugar levels in patients with a history of burn injury and diabetes, as well as the potential for infections to precipitate DKA. Timely intervention, including incision and drainage, can lead to successful resolution of symptoms and improved outcomes.

## Introduction

Necrotizing fasciitis (NF) is an infrequent and severe infection affecting the integumentary system and underlying subcutaneous tissue, characterized by the fast advancement of necrosis in the soft tissues. The occurrence of this condition is most often seen in elderly individuals who have an impaired immune system and are experiencing infections caused by a combination of aerobic and anaerobic microorganisms [1]. A significant proportion of individuals diagnosed with NF exhibit comorbidities such as gout, peripheral artery occlusive disease, myelodysplastic syndrome, liver cirrhosis, and other immunosuppressive diseases. Diabetes mellitus (DM) is often seen as a prevalent comorbidity in patients with NF. This is due to the fact that individuals with diabetes often have compromised cutaneous wound healing and heightened vulnerability to infections, factors that might potentially impact the progression of soft-tissue infections. Therefore, it is justifiable to hypothesize that this persistent and incapacitating ailment has a role in the heightened severity of NF [2]. Diabetic ketoacidosis (DKA) is a prevalent acute hyperglycemic emergency often seen in individuals diagnosed with DM. DKA occurs as a result of either a complete absence or inadequate levels of insulin, leading to an increase in counter-regulatory hormones. This condition typically manifests as a combination of hyperglycemia, metabolic acidosis, and ketosis (elevated ketone levels in the blood or urine, with a serum ketone concentration exceeding 3.0 mmol/l). Additionally, DKA is often accompanied by varying degrees of reduced circulatory volume [3]. Various infections including pneumonia, urinary tract infection, gastroenteritis, and sepsis are known to trigger episodes of DKA. The presence of infection further disrupts glycemic control and exacerbates the underlying insulin deficiency, leading to the development of ketosis and metabolic acidosis characteristic of DKA [4]. We present a case of a patient with a history of burn injury and poorly controlled diabetes who developed a post-auricular abscess, initially presumed to be a simple abscess, which ultimately led to the diagnosis of NF.

## Case presentation

**Figure 1:**
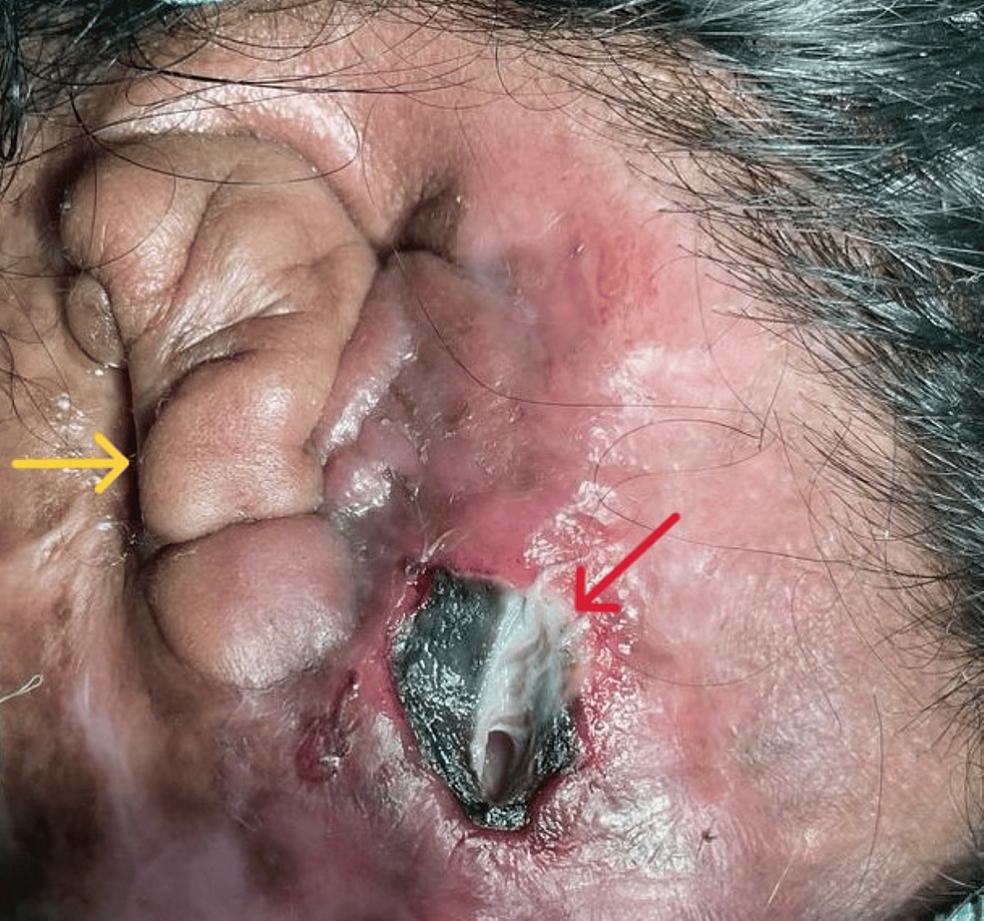
Photograph showing the area of necrotizing fasciitis with skin discoloration (marked by red arrow) and post-burn contracture of pinna (marked by yellow arrow).

A 45-year-old female with a history of burn injury 20 years ago and poorly controlled diabetes presented with a post-auricular abscess. The abscess was located near the site of the previous burn injury and therefore was believed to have developed as a result of thick scar tissue. The patient was on oral hypoglycemic medication for diabetes management. Upon examination, a post-burn contracture was observed on the left side of the face extending up to the neck with contraction of the pinna, resulting in thick scar tissue. It was now manifesting as infective cellulitis with the presence of a left post-auricular abscess (Figure 1).

On admission, the patient exhibited signs and symptoms of DKA, including hyperglycemia, metabolic acidosis, and ketonuria. The Glasgow Coma Scale (GCS) score of E3V4M5, indicating impaired consciousness, along with a blood pressure reading of 90/60 mmHg, blood sugar levels exceeding 450 mg/dl, the presence of 4+ urinary ketone bodies, and hypokalemia were observed (Table 1).

**Table 1:** Investigation profile of the patient on the day of admission Hb: Hemoglobin; TLC: Total Leucocyte Count; RBS: Random Blood Sugar; HbA1c: Hemoglobin A1C

Lab Parameters	Observed Value	Normal Range
Hb	12 gm%	13-17 gm%
TLC	20400 cells/cu mm	4000-10000 cells/cu mm
Platelets	2.57 Lakhs/cu mm	1.5-4.1 Lakhs/cu mm
Urea	33 mg/dL	19-43 mg/dL
Creatinine	0.6 mg/dL	0.66-1.25 mg/dL
Sodium	137 mmol/L	137-145 mmol/L
Potassium	3.5 mmol/L	3.5-5.1 mmol/L
Uric acid	4.2 mg/dL	3.5-8.5 mg/dL
RBS	450 g/dL	90-140 g/dL
HbA1c	12%	<5.7%
Urine sugar	++	Nil
Urine albumin	+	Nil
Urine ketones	++++	Nil

Management and outcome

**Figure 2:**
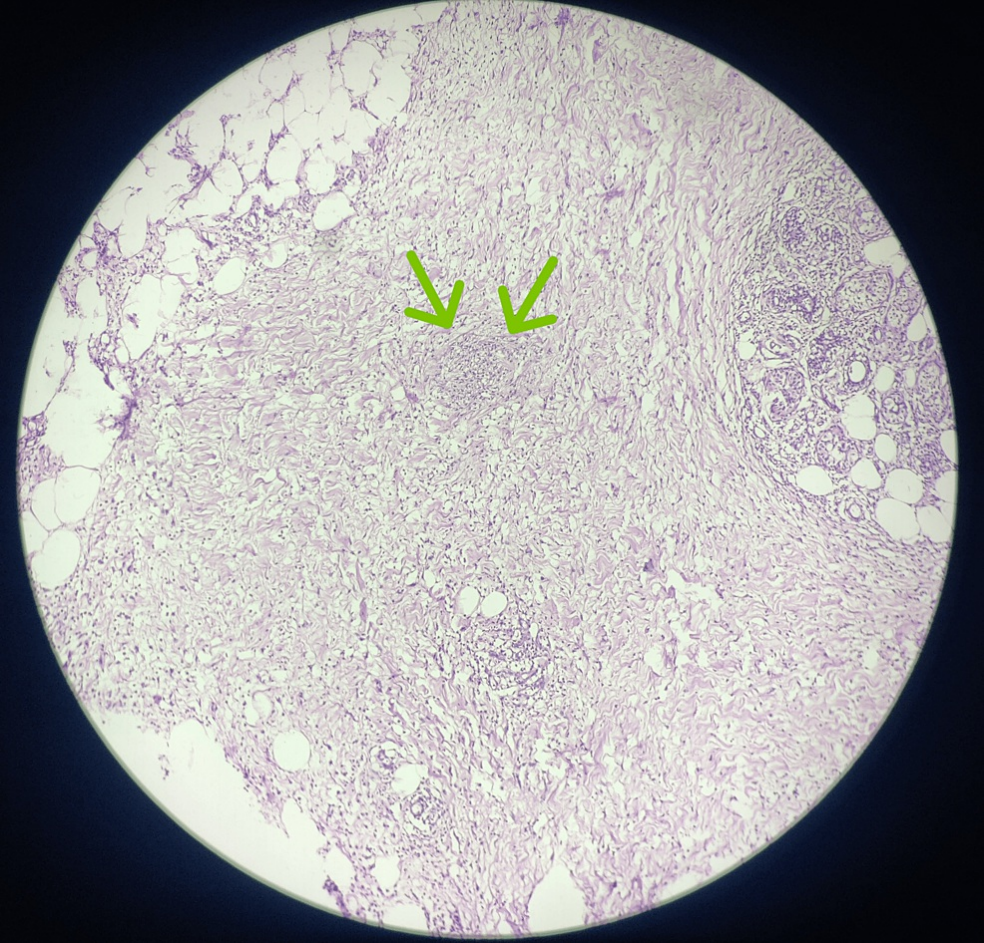
Photomicrograph of histopathological specimen at low power (4X) view showing necrotic fascial tissue with inflamed adipose tissue and micro-abscess formation (marked by green arrows).

**Figure 3:**
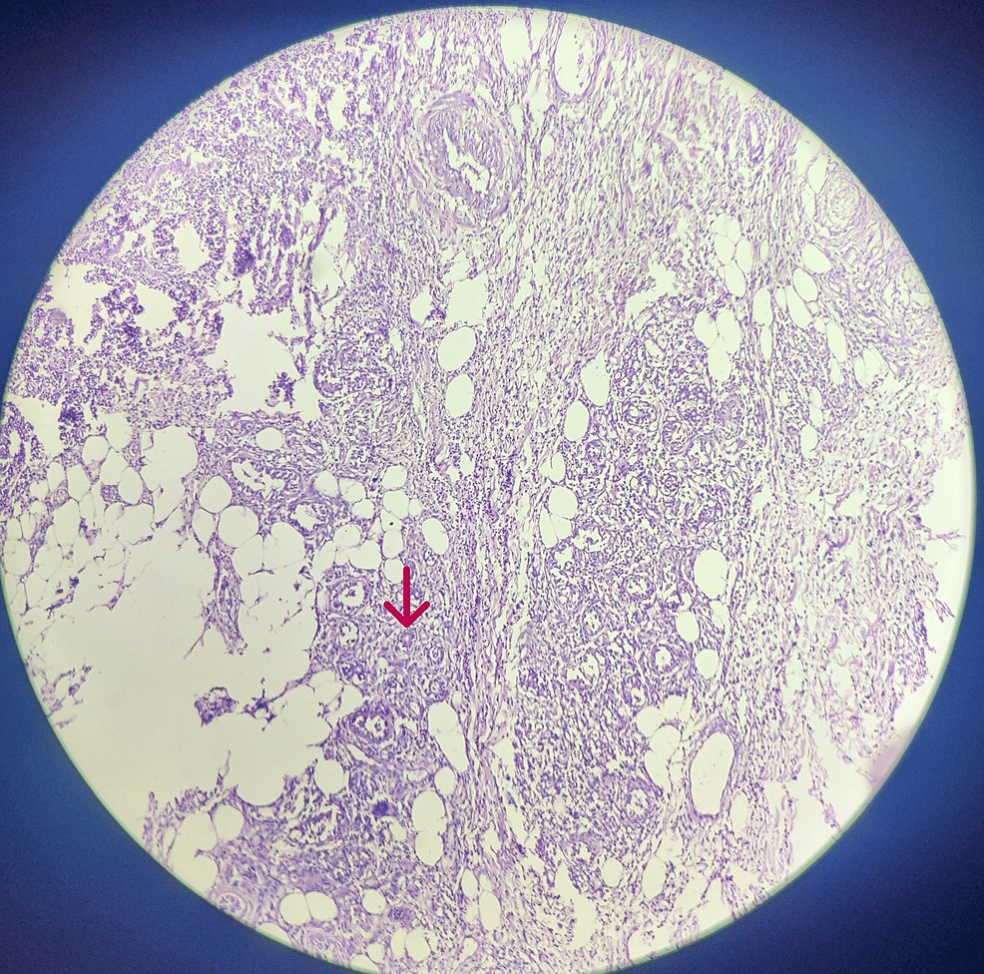
Photomicrograph of histopathological specimen at low power (4X) view showing necrotic fascial tissue with evidence of a thrombosed blood vessel (marked by red arrow). There is extensive inflammatory infiltrate scattered across.

The patient received prompt and appropriate management, including adequate hydration, intravenous antibiotics (ceftriaxone and sulbactam, amikacin, metronidazole), and insulin therapy to correct the ketoacidosis. Ultrasonography (USG) of the neck revealed the presence of a heterogeneous hypoechoic collection, spanning the cutaneous, subcutaneous, and intramuscular planes in the left post-auricular and mastoid region. The collection measured approximately 10-15 cc in volume with multiple calcific foci within the collection. Additionally, extensive cellulitic changes were observed in and around the posterior aspect of the left neck, giving rise to a cobblestone appearance. Under ultrasound guidance, abscess drainage was performed, resulting in the extraction of approximately 2-3 cc of yellowish, thick aspirate using a 16-gauge needle. Despite initial treatment, the abscess continued to grow, with increasing insulin requirements. Due to the worsening clinical condition and suspicion of a deeper infection, the patient underwent incision and drainage of the abscess. During the procedure, aggressive debridement was performed, revealing necrotic tissue consistent with NF. Cultures obtained from the debrided tissue confirmed the presence of a polymicrobial infection. A histopathological diagnosis of NF was made (Figures 2-3). Broad-spectrum antibiotics were initiated, targeting the identified pathogens.

**Figure 4:**
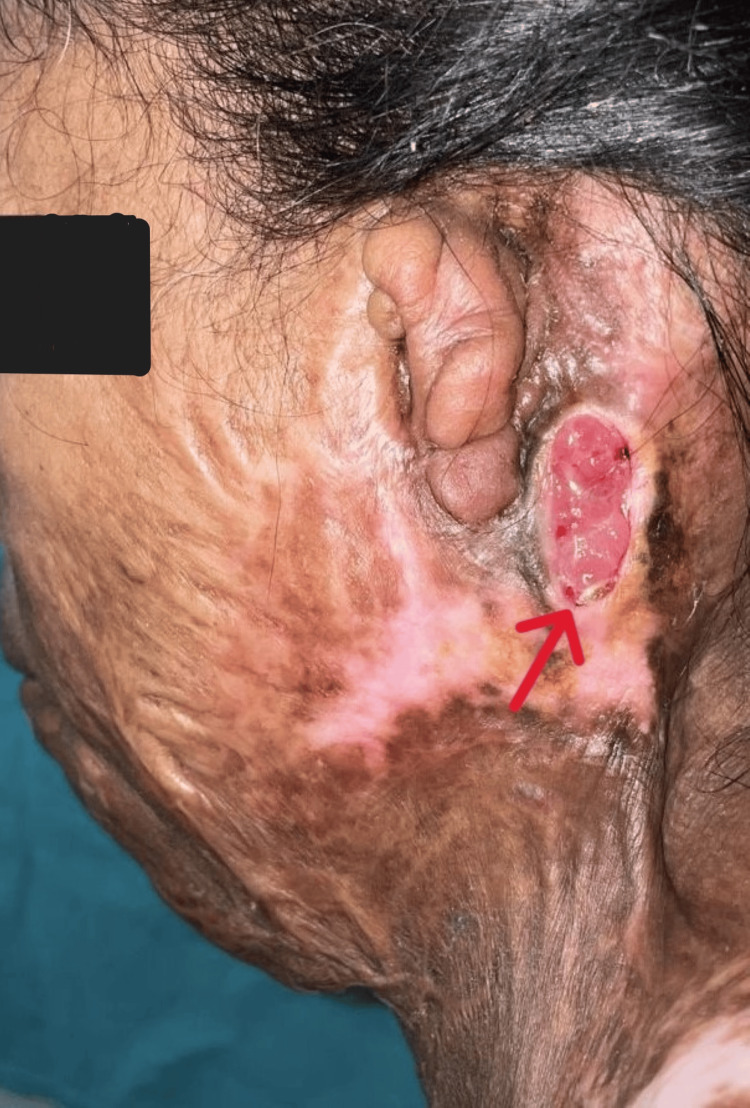
Photograph showing the healed area of necrotizing fasciitis (marked by red arrow) with granulation tissue.

Following the procedure, the patient's symptoms improved, and there was a rapid reversion of ketoacidosis. Serial monitoring of blood sugar levels showed a steady decline, and the patient's condition stabilized. The patient was discharged with appropriate wound care instructions and a revised diabetes management plan (Figure 4).

## Discussion

NF is an infrequent but potentially lethal illness that affects the subcutaneous tissue and fascia. It is a well-recognized condition. In the early stages, distinguishing NF from cellulitis and other superficial skin infections might provide challenges [5]. The described condition is characterized by a notably aggressive and infectious process that involves many types of microorganisms and mostly affects soft tissues. This condition poses a significant danger of fast dissemination across both superficial and deep fascial planes, as well as muscle layers [6]. The present case highlights the complex interplay between a history of burn injury, previous ear surgery, poorly controlled diabetes, and the development of a post-auricular abscess leading to DKA. The patient's medical history, including a burn injury and ear surgery in childhood, likely contributed to the formation of scar tissue in the affected area. Scar tissue resulting from burns can provide an environment conducive to the development of infections, leading to abscess formation and, in rare cases, NF.

The historical roots of NF may be traced back to ancient civilizations, as shown by the documentation of comparable pathological diseases by Hippocrates around the fifth century BC [7]. The name "necrotizing fasciitis" was first used by Wilson in 1952 to describe the primary characteristic of the illness, which involves the necrosis of the fascia and subcutaneous tissue, while the underlying muscle is mostly unaffected [7,8]. Cervical necrotizing fasciitis (CNF) is a very uncommon condition, mostly originating from dental infections, and may be further aggravated by the development of mediastinitis, leading to a significantly elevated fatality rate [6]. If not properly detected and treated, it has the potential to swiftly advance to systemic toxicity and can result in fatality. Once there is suspicion, care should include prompt resuscitation, early surgical debridement, and the introduction of broad-spectrum intravenous antibiotics [7]. In the present case, upon examination, a post-burn contracture was observed on the left side of the face extending up to the neck with contraction of the pinna, resulting in thick scar tissue, and was manifesting as infective cellulitis with the presence of a left post-auricular abscess.

Individuals diagnosed with NF may exhibit constitutional symptoms associated with sepsis, such as fever, tachycardia, altered mental state, and diabetic ketoacidosis. Alternatively, they may present with indications of skin inflammation, which might facilitate a relatively simpler diagnosis [5]. Certain systemic illnesses, such as diabetes, have the potential to decrease the patient's immunological competence, which may contribute to an increased susceptibility to certain health disorders. In the current scenario, the individual was prescribed oral hypoglycemic medicine for the purpose of managing her diabetes. On admission, the patient exhibited signs and symptoms of DKA, including hyperglycemia, metabolic acidosis, and ketonuria. The Glasgow Coma Scale (GCS) score of E3V4M5, indicating impaired consciousness, along with a blood pressure reading of 90/60 mmHg, blood sugar levels exceeding 450 mg/dl, the presence of 4+ urinary ketone bodies, and hypokalemia were observed.

Individuals diagnosed with DM exhibit increased vulnerability to infections as a consequence of many contributing variables. The aforementioned factors include a diminished migratory capacity of neutrophils, decreased phagocytic functionality, compromised humoral immune response, heightened adhesion of microbes to diabetic cells, neuropathy, and microangiopathy. These underlying mechanisms contribute to a compromised immune response, making individuals with DM more vulnerable to infections. Additionally, the frequency of medical interventions in DM patients increases the risk of bacteremia, further increasing their susceptibility to infections. Moreover, complications associated with DKA can impair leukocyte function and exacerbate the severity of infection [9]. In their study, Wang et al. put out the proposition that prognosis and clinical result in a preclinical model of bacterial infection might be strongly influenced by the type of the pathogen (e.g., bacterial, viral) and the infection profile. Additionally, the levels of glucose have a significant impact on the fate of animals that are afflicted. In conclusion, the viability of animals afflicted with bacterial sepsis, particularly due to infection by *Listeria monocytogenes*, exhibited a significant decrease when subjected to gavage feeding. Conversely, when these animals were administered glucose intraperitoneally, all of them experienced mortality [10]. These findings highlight the intricate relationship between glucose levels, infection, and prognosis in animal models of bacterial infection.

An optimal therapeutic approach encompasses timely diagnosis (based on clinical observations, local microbiological examinations, blood culture, and, if warranted, histopathology), initiation of broad-spectrum antibiotic treatment at the earliest feasible opportunity, subsequent adjustment of antibiotic regimen based on antibiogram findings, stabilization of essential physiological functions, and, when feasible, addressing and managing underlying factors that may contribute to the condition [6]. In the present case, the patient received prompt and appropriate management, including adequate hydration, intravenous antibiotics, and insulin therapy to correct the ketoacidosis. USG of the neck revealed the presence of a heterogeneous hypoechoic collection, spanning the cutaneous, subcutaneous, and intramuscular planes in the left post-auricular and mastoid regions.

Early and complete operative debridement is the cornerstone of treatment for necrotizing soft tissue infection (NSTI) [11,12]. Inadequate debridement has been associated with a seven-fold increase in mortality [10], emphasizing the criticality of timely intervention. Delay in surgical intervention has also been linked to a significantly higher likelihood of death [13]. It is important to recognize that the area of necrosis can extend beyond the apparent external appearance of the skin, attributed to thrombosis of dermal capillary beds preceding skin necrosis. Therefore, the true extent of tissue involvement may be underestimated based solely on external findings. Thorough and prompt debridement is essential to eliminate necrotic tissue, eradicate the source of infection, and prevent further dissemination, ultimately improving patient outcomes and minimizing complications associated with NSTI [14].

Early incisional drainage and debridement in diabetic patients with abscesses can lead to improved prognosis. In the present case, these procedures were performed promptly and successfully reduced the size of the large abscess. While surgical procedures can be associated with complications such as bleeding, bacteremia, fistula formation, and poor cosmetic outcomes, no complications were observed in our case [15]. The diagnosis of NF is challenging as initial clinical findings usually resemble a simple abscess. Thus, surgical intervention, including incision and drainage with aggressive debridement, is often necessary to diagnose and manage NF effectively.

## Conclusions

Post-burn necrotizing fasciitis (PBNF) may be difficult to diagnose in its early stages due to scars and contractures and mimics an ordinary abscess. This case emphasizes the importance of a high index of suspicion and vigilance in monitoring blood sugar levels in patients with suspected NF and diabetes. Infections, even seemingly minor ones, can have serious consequences in the form of increased risk of mortality and precipitation of DK. Timely intervention, including incision and drainage, along with aggressive debridement, led to favorable outcomes in a case of PBNF in a diabetic patient and helped to reverse DKA.
